# Biomechanical Testing of Two-Unit Bridges and a Comparison of Replacement Retention Depending on a Cementation Medium, Replacement Position, and Gap Size

**DOI:** 10.3390/jfb13040286

**Published:** 2022-12-08

**Authors:** Alena Findrik Balogová, Viktória Rajťúková, Ľuboš Chromý, Andrej Somoš, Gabriela Ižaríková, Radovan Hudák

**Affiliations:** 1 Department of Biomedical Engineering and Measurement, Faculty of Mechanical Engineering, Technical University of Košice, 040 01 Košice, Slovakia; 2 Clinic of Pneumology and Phthisiology, L. Pasteur University Hospital Košice, 040 11 Košice, Slovakia; 3 Department of Applied Mathematics and Informatics, Faculty of Mechanical Engineering, Technical University of Košice, 040 01 Košice, Slovakia

**Keywords:** biomechanical testing, cobalt-chrome, dental cement, dental replacements, gap size

## Abstract

Dental replacements are placed between the abutment teeth. The exceptions are two-unit bridges, as they are supported by a single tooth prepared only on one side of the missing tooth. The presented study deals with an analysis of a pressure force action on two-unit bridges placed in the frontal part (20 samples), where the pressure action is lower, and in the distal part (20 samples), where the pressure action is higher. A CAD program by 3Shape was used for digital designing with two different gap settings, 10 μm (20 samples) and 30 μm (20 samples). Two-unit bridges were attached to the prepared tooth using two types of dental cement (20/20 samples), which were selected for their physical and bioactive properties. All two-unit bridges (a total of 80 samples) were fabricated from CoCr alloys on Mlab cusing R by applying the Selective Laser Melting (SLM) technology. Mechanical testing was performed using the Inspekt5 table blue. The obtained data were used to verify the hypotheses—a difference between both types of cement (A ≠ B), a difference between the frontal and distal two-unit bridges (F ≠ D) and a difference between the gap sizes (10 ≠ 30). To confirm the given theories, data were statistically evaluated using the F-test and subsequent *t*-tests. The resulting *p*-value was compared with the level of significance (α = 0.05). A statistical evaluation revealed a significant difference between the compared groups; however, no explicit correlation between the individual groups of specimens was identified.

## 1. Introduction

The development of novel technologies affects all of the spheres of human activities, including medicine and, in particular, stomatology. Innovative technologies facilitate, for example, more accurate processing of input data (processing of imprints and models) using scanning systems, as well as more efficient manufacturing technologies that improve production planning and automation. A great advantage of digital technologies is the visualisation of a planned product and the possibility to perform echo checks in the system.

Cobalt-chrome alloys (CoCr) are the materials that are widely used in dentistry. Despite the fact that novel materials are being developed, this particular material still ranks among the key materials used in the production of dental replacements. Cobalt- and chrome-based alloys are largely used for various orthopaedic implants and in the production of metal bases for fixed dental prostheses due to their excellent mechanical properties, such as high resistance to corrosion and wear, and good biocompatible properties. The chemical composition of Co-Cr dental alloys includes Co in 53–67%; Cr in 25–32%; Mo in 2–6%; and small amounts of W, Si, Al, and other elements. Chrome, molybdenum, and wolfram are added to increase the strength [1]. Due to a relatively high amount of Cr, a thick passive layer of Cr_2_O_3_, 1–4 nm thick, is formed on the surface. This layer determines such a high resistance to corrosion [1,2,3].

At present, the structures made of CoCr are typically produced by casting. This process requires several manual steps to be carried out in the manufacturing process. There is a novel method of CNC milling into metal blocks. Although this method exhibits high accuracy, its disadvantage is a significant amount of waste or unused material. The latest, albeit the least used technology, is the manufacturing using Selective Laser Melting (SLM) from metal powder. Layers of metal powder are melted into a 3D model using a computer-controlled laser. Advantages of SLM, when compared to conventional methods, include the possibility of manufacturing customised complex models and parts with a dense structure and predefined surface roughness. This facilitates the fabrication of robust as well as very fine elements while the quantities of waste materials are negligible as the powder is recyclable [4,5,6]. 

Fabrication of dental replacements and the efficiency of their use depend not only on a production method and the material used but also on a device design. In addition, marginal and internal gaps rank among the most important aspects that determine the expected service life and safety of dental replacements. If there is a microscopic crack or leak near the marginal gap, cement begins to dissolve [7], and this could lead to the secondary accumulation of plaque, the formation of caries, and gingivitis [7,8]. Moreover, the inner space between the replacement and the prepped tooth affects the retention and occlusion. If the internal gap is too wide or narrow, the filling may become displaced, or it may lead to malocclusion or imperfect insertion of the replacement [9]. Therefore, marginal and internal gaps are the critical factors that determine the success of the fabrication of dental replacements [10,11,12,13,14,15].

In the manufacturing of dental replacements, in particular dental bridges, there are two types of two-unit bridges. Implants located in the frontal area—the impact of the force exerted during a bite decreases towards the frontal part of the mouth. However, the bite forces are still exerted outside the bridge axis, and hence more pressure is applied to the supporting teeth. Implants located in the distal area—the bite forces are much higher in the posterior part of the mouth. As a result, the bite forces exerted on the supporting teeth are extremely high. After a prolonged period of time, the supporting teeth may loosen, and this may jeopardise the whole restoration [16].

Nowadays, additive technology for the production of metal dental prostheses is increasingly being used in the dental field. The presented study deals with the testing of samples produced by the SLM technology from the cobalt-chromium alloy [17,18,19,20]. 

The purpose of the submitted study was to apply the SLM method in order to manufacture two types of two-unit bridges made of CoCr material. These two-unit bridges were attached to a stump made of PEEK (with properties that are similar to those of the human bone) and then subjected to compression while monitoring the resistance of the individual cement types to the applied force. The cement types were selected based on their properties [21]. The impact of the post-processing in the SLM manufacturing (annealing) process was evaluated by comparing the annealed and non-annealed two-unit bridges using GOM INSPECT software (Carl Zeiss AG, Jena, Germany). The purpose was to use the results of the performed mechanical tests to identify the effects of the cement types, positions of the two-unit bridges, and gap sizes, as these parameters affect the aesthetics of the dental replacement attachment.

## 2. Materials and Methods

**Figure 1:**
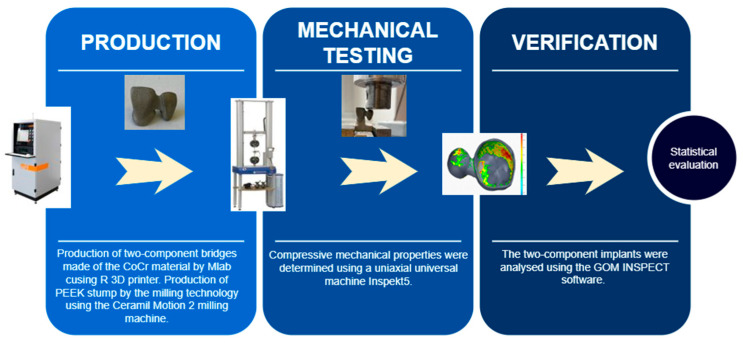
Workflow of the present study.

The methodology of the study consisted of three main steps (Figure 1): production (designing and modelling two-unit bridges and a stump; fabrication of bridges and test stumps), mechanical testing and verification (statistical evaluation of results).

### 2.1. Designing and Modelling Two-Unit Bridges

The models of the two-unit bridges were designed using a plaster model, which was scanned with a 3Shape D700 dental scanner (3Shape, Copenhagen, Denmark). The obtained data were subsequently used to design the model. The selected preparation method was the attachment onto a plate, with a convergence angle of 3–8° and a cervico-occlusal reduction of >3 mm, proposed by the software as the optimal values.

The model was designed in the CAD programme using 3Shape software (3Shape, Denmark), while the necessary parameters were set in the initial stage of the modelling. One of the key parameters for the submitted study was to create a space for cement, the so-called “reduction” or a “gap“, the sizes of which were set to 0.01 mm (10 μm) and 0.03 mm (30 μm). The modelling outputs were two types of bridges: frontal bridges (Tooth 11—a crown; Tooth 12—an intermediate component (pendes)) and distal two-unit bridges (Tooth 24—a crown; Tooth 25—pendes).

### 2.2. Designing and Modelling Test Stumps

After modelling the frontal and distal two-unit bridges with two different types of gaps, the scanned plaster model was used to design the stumps onto which the CoCr bridges were attached. In the 3Shape environment, the redundant areas of the dental arch were cut off, and the stumps were generated for the frontal (20 samples) and distal (20 samples) two-unit bridges. Subsequently, the shapes of the stumps were adjusted to meet the requirements of the compressive testing.

### 2.3. Material

The test specimens (two-unit bridges) were fabricated using the Starbond Easy CoCr Powder (Scheftner Dental, Mainz, Germany), consisting of Co in 61%; Cr in 27.5%; W in 8.5%; Si in 1.6%; and C, Fe, and Mn contained in less than one percent. The powder grain size ranged from 10 μm to 30 μm. This metal powder is used in the production of dental replacements manufactured by Selective Laser Melting (SLM) technology. Table 1 contains the data on the mechanical properties of this powder.

Three different materials were considered for the fabrication of the test stumps, i.e., PEEK, CoCr, and zirconium. Table 2 presents the mechanical properties of the individual biological dental materials; based on these properties, the material selected for the final fabrication was PEEK, as its mechanical properties were the most similar to the properties of a native bone. 

### 2.4. Fabrication of Bridges and Test Stumps

The modelled test specimens were imported to CAMbrigde software (3Shape, Denmark), in which the model positions were determined and the supporting structures were designed; the models were designated with numbers and placed on the build plate. Subsequently, data were generated and imported to the Concept Laser software (GE Additive, Boston, MA, USA) in the Mlab cusing R 3D printer (GE Additive, USA).

The PEEK stumps were produced by the milling technology using the Ceramil Motion 2 milling machine (Amann Girrbach, Koblach, Austria).

### 2.5. Annealing and Its Effects on the Specimens

The production of the two-unit bridges was followed by post-processing which comprised the annealing of the build plate together with the produced models (max. 1150 °C). The annealing process was followed by the removal of the supporting structures and surface finishing of the two-unit bridges.

One of the key parameters monitored in the study was the creation of gaps sized 10 μm (20 samples) and 30 μm (20 samples). These dimensions were small, and in order to ensure the relevancy of the results, it was necessary to exclude variables in the post-processing stage, such as annealing and accuracy of the production of the two-unit bridges. The purpose of annealing was to remove the strain that was present in the supporting structure in order to avoid the model deformation during its removal off the build plate. For the purpose of verification of the effects of annealing on the shapes of the two-unit bridges, the testing was carried out with 10 two-unit bridges.

### 2.6. Mechanical Testing

Compressive strength is the value of stress at which a specimen becomes completely damaged. This value was obtained experimentally based on the results of the compression tests. Compressive strength is defined as the force (*F*) acting on the area (*A*) at the instant of the material destruction or until the predefined conditions are achieved. The basic definition describes stress as follows:

*σ* = *F*/*A* [MPa] (1)

Compressive mechanical properties were determined using a uniaxial universal machine, Inspekt5 table blue (Hegewald & Peschke, Nossen, Germany). This machine uses a maximum load of 5 kN. The measuring range of the device was compliant with DIN EN ISO 7500-1, ASTM E4.

**Figure 2:**
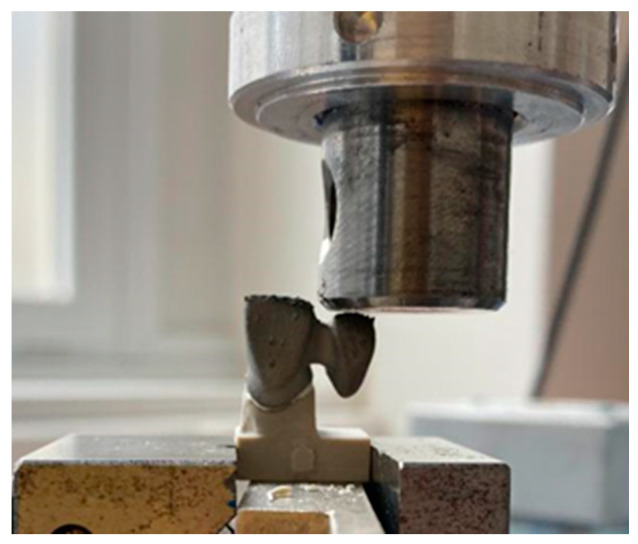
Application of the loading force onto the test specimen.

Each specimen was exposed to compressive loading while the cross-beam was moving at a speed of 1 mm/s; the force was applied until the crown loosened from the stump, accompanied by a reduction in the force magnitude (ISO 4049:2019). The force was only applied to the intermediate component in order to create the necessary lever effect (Figure 2). Prior to the testing, the stumps and the two-unit bridges were sand-blasted. The total number of tested two-unit bridges was 80 pieces that were made of CoCr and attached to the PEEK stumps.

These mechanical tests were evaluated statistically in order to assess the effects of the cement types and positions of the two-unit bridges and to compare the created gaps in the two-unit bridges.

## 3. Results

Within the evaluation of all the tests and experiments, the individual test specimens were systematically designated based on their positions and gap sizes in order to make them easily distinguishable (Table 3).

### 3.1. Preparation of Dental Test Specimens

**Figure 3:**
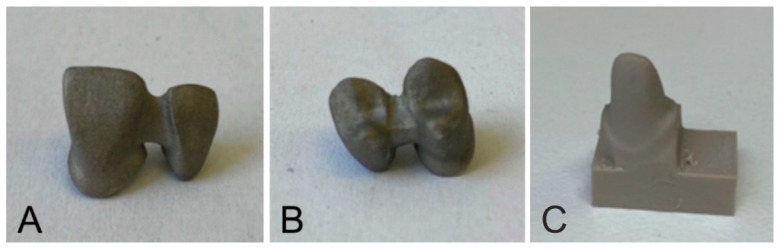
Two-unit bridges made of CoCr; (**A**)—frontal; (**B**)—distal; (**C**)—a PEEK stump.

Of the two-unit bridges, 80 specimens were fabricated using two types of cement, in particular, zinc polycarboxylate and zinc phosphate cement. Each group contained 40 frontal and 40 distal bridges. These groups of specimens included 20 specimens with gaps sized 10 µm and 20 specimens with gaps sized 30 µm. The specimens were manufactured by the additive manufacturing technique, Selective Laser Melting (SLM), using the Starbond Easy CoCr Powder (Scheftner Dental, Germany) consisting of Co in 61%; Cr in 27.5%; W in 8.5%; Si in 1.6%; and C, Fe and Mn representing less than one percent. The powder grain size ranged from 10 μm to 30 μm. The produced specimens were sand-blasted. The figures below show the produced frontal and distal two-unit bridges (Figure 3A,B). For the individual types of two-unit bridges, 80 test stumps were fabricated from the PEEK material and subjected to milling using the Ceramill Motion 2 milling machine (Amann Girrbach, Austria) (Figure 3C).

The production of the test specimens and stumps was followed by the cementing process, which consisted of applying the cement, positioning the two-unit bridges onto the stump, and removing the excess cement.

The prepared test specimens were left to harden for 60–90 min in order to facilitate complete cement hardening.

### 3.2. Effects of Annealing on the Accuracy of the Two-Unit Bridges

For the purpose of verification of the effects of annealing on the implant shape, 10 two-unit bridges were subjected to testing. The following comparisons were made in the GOM INSPECT environment (Carl Zeiss AG, Jena,, Germany):

- The modelled two-unit bridges (Nominal, N) and the model without annealing (Actual1, A1);
- The modelled two-unit bridges (Nominal, N) and the model after annealing (Actual2, A2);
- The model before annealing (Actual1, A1) and the model after annealing (Actual2, A2).

**Figure 4:**
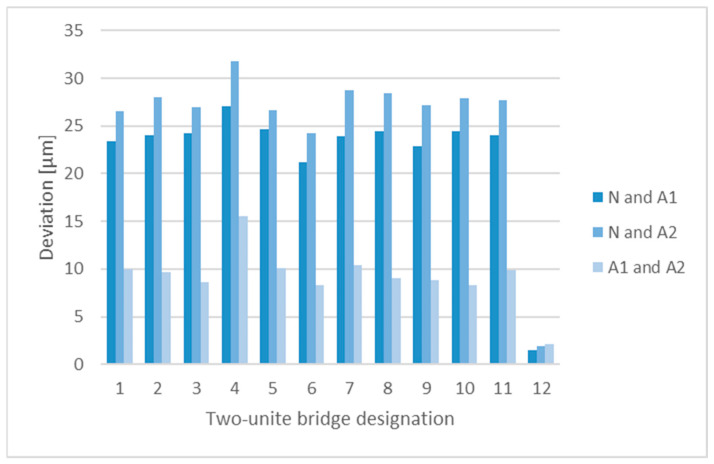
Dimensional deviations identified by comparing the Nominal and Actual models.

**Figure 5:**
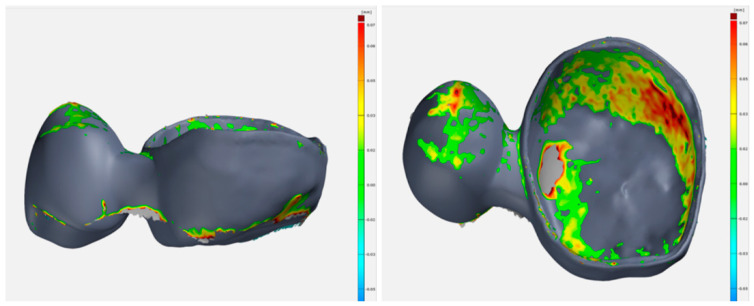
Nominal and actual overlapping in the GOM INSPECT environment.

In order to examine the production accuracy, 100 two-unit bridges were analysed using the GOM INSPECT software (Carl Zeiss AG, Jena,, Germany); the comparisons were made between the designed models (N) and the final products (A)—after annealing, removal of the supporting structure, surface finishing, and sand-blasting. The resulting dimensional deviations are shown in Figure 4. The overlay of Nominal and Actual in the GOM INSPECT environment are shown in Figure 5.

### 3.3. Evaluation of Mechanical Compressive Testing

**Figure 6:**
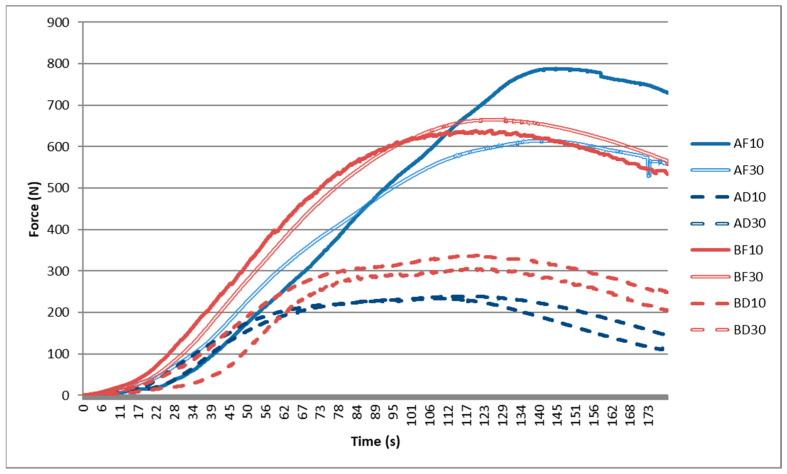
Curves of mechanical loading of the two-unit bridges.

Based on the mechanical testing of the printed specimens, all the data was processed in the form of graphs. The graphs represented the values of the force magnitude changing over time and the average curves of the groups of specimens (Figure 6).

The individual measurements indicated that in order to achieve displacements of the two-unit bridges as a result of the applied compression (cement loosening), for the frontal specimens, the required force was approximately 1.5–2-fold higher than the force required for loosening cement on the distal specimens. Therefore, it may be concluded that a bridge in the distal position is more resistant than the one in the frontal position.

### 3.4. Statistical Evaluation of the Effect of a Specimen Type on Strength

The obtained data were used to verify the following hypotheses: (i) There is a difference between both types of cement (A ≠ B); (ii) there is a difference between the frontal and distal two-unit bridges (F ≠ D); (iii) there is a difference between the gap sizes (10 ≠ 30).

A statistical evaluation of the data was made to confirm these theories. The two sets were compared using the F-test and, subsequently, the *t*-tests. The resulting *p*-value was compared to the significance level (α = 0.05). If the *p*-value ˂ α, then the null hypothesis on the equality of mean values are rejected.

### 3.5. Comparison of the Cement Types

**Figure 7:**
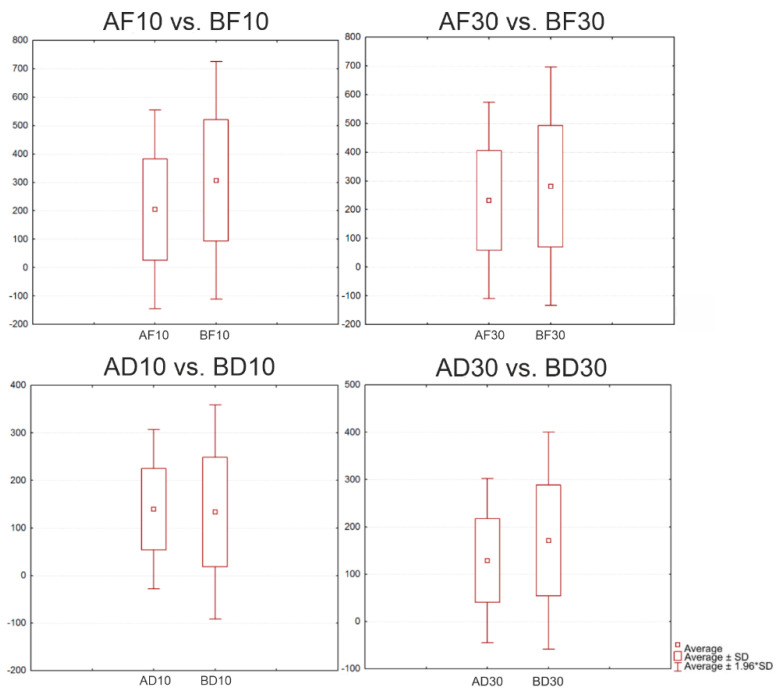
Boxplots for the distal position with Cement A and Cement B and two gap sizes.

The evaluation of the cement types included a statistical comparison of the frontal and distal specimens (Figure 7). The following groups were compared: AF10 vs. BF10; AF30 vs. BF30; AD10 vs. BD10; and AD30 vs. BD30 (Table 4).

In each of the comparisons, the *p*-value was zero; this confirmed a statistically significant difference between the individual groups of specimens.

### 3.6. Comparison of the Positions of the Two-Unit Bridges

**Figure 8:**
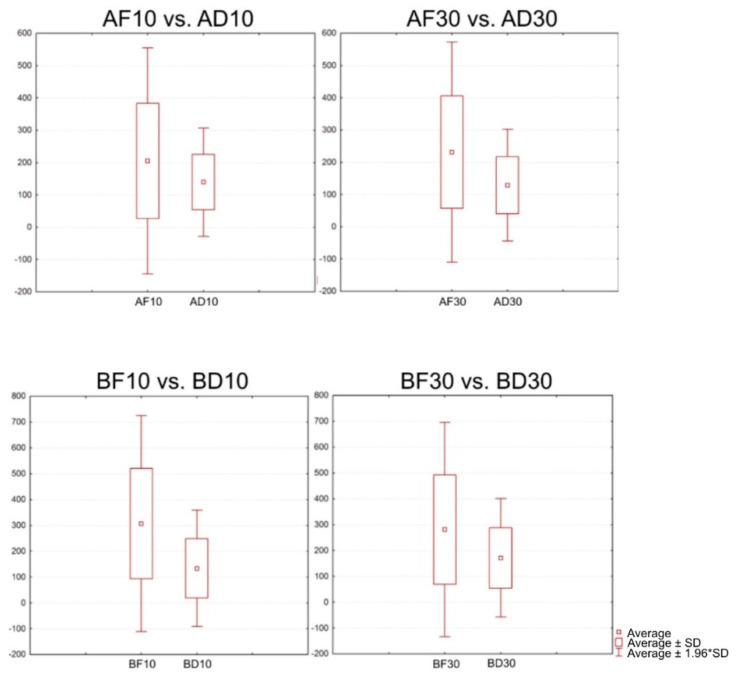
Boxplots for a comparison of the positions of the two-unit bridges.

The following statistical comparison was made for the frontal and distal types of specimens (Figure 8). The following groups were compared: AF10 vs. AD10; AF30 vs. AD30; BF10 vs. BD10; and BF30 vs. BD30 (Table 5).

In each of the comparisons, the *p*-value was zero; this confirmed a statistically significant difference between the individual groups of specimens.

### 3.7. Effects of the Sizes of Gaps in the Two-Unit Bridges

**Figure 9:**
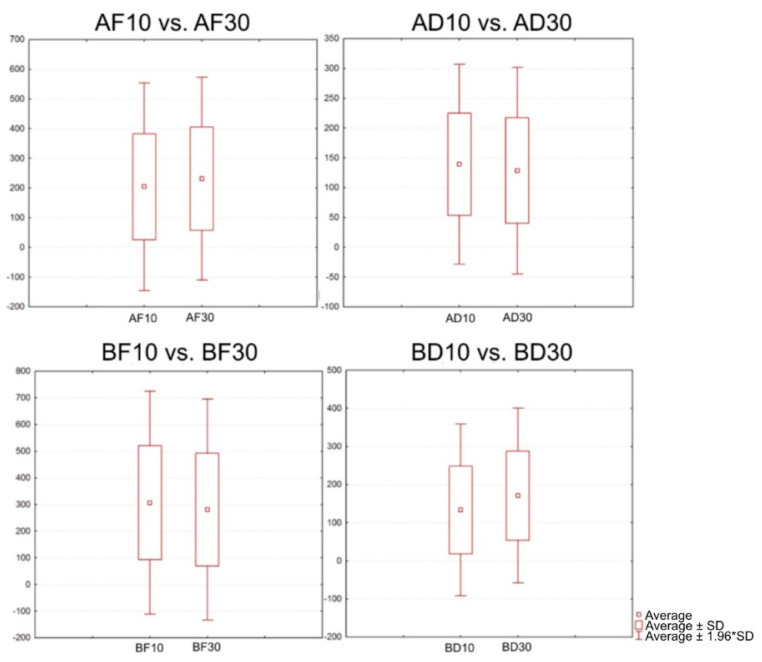
Graphical representation of the comparison of the gap sizes in the two-unit bridges.

It was assumed that there was a difference between the individual sizes of gaps in the two-unit bridges (Figure 9); therefore, the following groups of specimens were compared: AF10 vs. AF30; AD10 vs. AD30; F10 vs. BF30; and BD10 vs. BD30 (Table 6).

The individual comparisons have confirmed that there were statistically significant differences between the groups of specimens.

For Cement A, the average force required to break the AF10 samples was 847 N, and for the AF30 samples, it was 634 N. Based on the findings, we can conclude that with the frontal location and a gap of 10 μm, a greater force is required to release the two-unit bridge from the stump. The average force required to break the AD10 samples was 239 N, and for the AD30 samples, it was 249.97 N. Based on the findings, we can conclude that the distal location with a gap of 30 μm requires a greater force in order to release the two-unit bridge from the stump.

The same testing was done with Cement B. For the BF10 samples, the average force was 651 N, and for the BF30 samples, it was 672 N. For the BD10 specimens, the average force was 315 N, while for the BD30 specimens, it was 345 N. No significant differences were found in this testing.

The obtained results indicated that the type of cement, the location, as well as the gap size affected the average magnitude of the force exerted on the two-unit bridges; based on the aforementioned facts, we can conclude that the established hypotheses are valid.

## 4. Discussion

The presented study deals with the investigation into metal two-unit bridges produced by the SLM technology and their ability to withstand mechanical loading. Three-dimensional printing and digitisation are already becoming commonplace in dental laboratories. Various authors have carried out comparisons of grinding procedures used in the production of dental prostheses by applying conventional, subtractive, and additive techniques [18,19,20,21]. Based on the comparisons of their microstructures and mechanical properties, but also from an economic point of view, additive technology has been proven to be the ideal method for manufacturing dental prostheses [22,23,24,25,26].

Dental prostheses are placed between the abutment teeth, and the force is distributed over the entire surface of the dental prostheses. The exceptions are two-unit bridges, as the supporting tooth is prepared only on one side of the missing tooth; as a result, a greater force is applied to the prepared tooth. Current studies are preferably aimed at examining the loading exerted on bridges consisting of three and more units, whereas there are only a few similar studies that investigate the effects of forces exerted on cantilever two-unit dental bridges.

In this study, we focused on three main parameters that influence the resulting load on the prepared tooth in two-unit bridges—cement types, gap sizes, and positions. For a correct comparison of the results, it was necessary to identify the forces acting in the jaw during a physiological bite. The maximum bite force values were observed in the distal area, where they reached 580 N. In the frontal area, the bite force ranged from 180 to 320 N. With the use of dental prostheses, the forces acting during biting reduced in the distal area to approx. 220 N and in the frontal area to approx. 111 N [25,26,27,28,29]. Based on the comparison of the results achieved in the studies, the established hypotheses were confirmed.

Even though there are multiple factors that should be taken into account when selecting a cement type, when it comes to retention, the dominating ones are the mechanical properties of the prepped tooth, especially when water-based cement types are used. In such cases, water-based cement types may be used. Due to the fact that these cement types are easy to handle and have more favorable properties than the polymerising cement types, they may be used primarily when the surface lines of the preparation are sublingual, when it is impossible to perfectly regulate the gap fluid, or when it is impossible to achieve a completely dry environment [30,31].

In all of the available approaches to adhesive cementation, it is necessary to properly adjust the surface of dental tissues and of the cementation area. With glue applications, it is possible to achieve homogeneous dental cement by applying the most appropriate conditioning method for each group of dental materials, in particular metals, ceramics, and polymers [32]. 

Despite the frequent use of the additive manufacturing technique, conventional and subtractive methods of manufacturing dental prostheses are still used in the dental field. Therefore, it would be useful to make a comparison of two-unit bridges produced by conventional and subtractive technologies.

## 5. Conclusions

An optimal cementation model should be chosen while considering the filling (i.e., the properties of the filling material, marginal adjustment, a filling type, and surface finishing), the properties of cement (i.e., viscosity, biocompatibility, adhesive potential, solubility, and water consumption), colour stability, resistance to wear, working and setting properties, ability to seal, optical properties and radioactivity), as well as various clinical properties, such as occlusion, preparation type (retention, non-retention), humidity regulation, the material of the super-structure, support type (natural tooth structure: enamel, dentin, cement) or supporting implants (titanium, oxide ceramics), support mobility and surface roughness [33].

There are many available commercial brands of various types of cement from which a dentist must choose. These cement types, each one of them possessing different properties, must be scientifically examined. Tensile strength and compressive strength, in particular, are the properties that must be correctly measured before selecting a cement type. Tensile/compressive testing is normally carried out, but its results may sometimes appear contradictory due to differences in the test criteria [34].

Based on the performed experiments, tests, and the produced review of the mechanical properties of dental materials, the material that was eventually identified as the most appropriate for the production of stumps and as possessing the properties that are the most similar to those of dental tissues, is PEEK from which the stumps were milled. Onto these stumps, two-unit bridges with various gap sizes were attached using two different types of cement. The results of mechanical compressive testing of the two-unit bridges indicated that after comparing the individual cement types in all groups of specimens with zinc phosphate cement, the force that was required to break a bond between the bridge and the stump was on average 15% higher. Only with the distal specimens with a gap size of 0.01 µm the difference under compression was negligible [35]. 

A comparison of the positions of the two-unit bridges indicated a significant difference between the frontal and distal positions; in the frontal position, the force required to break the cement bond was higher in all of the cases. 

A comparison of gap sizes in the two-unit bridges indicated that their behaviour under mechanical compression was variable. A statistical evaluation revealed significant differences between the compared groups; however, no explicit correlation between the individual groups of specimens was identified.

In the presented study, the methodology for measuring the effect of pressure exerted on two-unit bridges manufactured by the additive manufacturing technique was established. In further studies, it would be appropriate to compare two-unit bridges made by conventional technology and two-unit bridges made by subtraction technology while applying the same methodology.

## Figures and Tables

**Table 1:** Mechanical properties of the Starbond material.

Mechanical Property	Value
Yield strength (Rp0.2)	760 MPa
Tensile strength	1090 MPa
Relative elongation at break	15%
Module of elasticity	225 GPa
Vickers hardness	425 HV 10
Type (DIN EN ISO 22674)	Class 5

**Table 2:** Comparison of mechanical properties of dental materials.

Material	Module of Elasticity [GPa]
Ceramill PEEK (Amann Girrbach, Austria)	4
Ceramill Zolid (Amann Girrbach, Austria)	>200
Starbond Easy CoCr Powder (Scheftner Dental, Germany)	225
Enamel	80
Dentin	18.6
Cortical bone	13.7
Spongy bone	1.37

**Table 3:** Designations used in the evaluation of the testing.

		Designation
**Cement Type**	Zinc polycarboxylate	A
	Zinc phosphate	B
**Position**	Frontal	F
	Distal	D
**Gap size**	10 μm	10
	30 μm	30

**Table 4:** Results of the *t*-test for the evaluation of the comparison of cement types.

Group 1 (S1)	Group 2 (S2)	Average S1	Average S2	t Value	*p*	Standard Deviation S1	Standard Deviation S2	F-Ratio Dispersion	P Dispersion
**AF10**	**BF10**	204.75	306.77	−25.94	0.00	178.44	213.32	1.43	0.00
**AF30**	**BF30**	231.63	281.3	−12.81	0.00	173.95	211.77	1.48	0.00
**AD10**	**BD10**	139.38	133.37	2.97	0.003	85.55	114.88	1.80	0.00
**AD30**	**BD30**	128.77	171.19	−20.45	0.00	88.40	117.04	1.75	0.00

T-Test for Independent Samples (data_5000); Note: The Variables Were Regarded as Independent Samples.

**Table 5:** T-test for a comparison of the positions of the two-unit bridges.

Group 1 (S1)	Group 2 (S2)	Average S1	Average S2	t Value	*p*	Standard Deviation S1	Standard Deviation S2	F-Ratio Dispersion	P Dispersion
**AF10**	**AD10**	204.75	139.38	23.36	0.00	178.44	85.55	4.35	0.00
**AF30**	**AD30**	231.63	128.77	37.27	0.00	173.95	88.40	3.87	0.00
**BF10**	**BD10**	306.77	133.37	50.61	0.00	213.32	114.88	3.45	0.00
**BF30**	**BD30**	281.29	171.19	32.18	0.00	211.77	117.04	3.27	0.00

T-Test for Independent Samples (data_5000); Note: The Variables Were Regarded as Independent Samples.

**Table 6:** T-test for a comparison of the positions of the two-unit bridges.

Group 1 (S1)	Group 2 (S2)	Average S1	Average S2	t Value	*p*	Standard Deviation S1	Standard Deviation S2	F-Ratio Dispersion	P Dispersion
**AF10**	**AD10**	204.75	231.63	−7.63	0.00	178.44	173.95	1.05	0.07
**AF30**	**AD30**	139.38	128.77	6.09	0.00	85.55	88.40	1.07	0.02
**BF10**	**BD10**	306.77	281.29	5.99	0.00	213.32	211.77	1.01	0.61
**BF30**	**BD30**	133.36	171.19	−16.31	0.00	114.88	117.04	1.04	0.19

T-Test for Independent Samples (data_5000); Note: The Variables Were Regarded as Independent Samples.

## Data Availability

Not applicable.
